# Combined Aerobic and Resistance Exercise in Walking Performance of Patients With Intermittent Claudication: Systematic Review

**DOI:** 10.3389/fphys.2019.01538

**Published:** 2020-01-08

**Authors:** Isabel Machado, Nelson Sousa, Hugo Paredes, Joana Ferreira, Catarina Abrantes

**Affiliations:** ^1^Department of Sports Science, Exercise and Health, University of Trás-os-Montes e Alto Douro, Vila Real, Portugal; ^2^Research Center in Sports Sciences, Health Sciences and Human Development (CIDESD), Vila Real, Portugal; ^3^Public Health Unit of Santo Tirso, ACES Grande Porto I-Santo Tirso/Trofa, Santo Tirso, Portugal; ^4^Department of Engineering, University of Trás-os-Montes e Alto Douro, Vila Real, Portugal; ^5^Institute for Systems and Computer Engineering, Technology and Science (INESC TEC), Porto, Portugal; ^6^Hospital of Senhora da Oliveira/EPE, Angiology and Vascular Surgery, Guimarães, Portugal; ^7^Life and Health Sciences Research Institute (ICVS), School of Health Sciences, University of Minho, Braga, Portugal

**Keywords:** peripheral arterial disease, intermittent claudication, combined aerobic and resistance training, walking performance, systematic review

## Abstract

**Background:** The short-term benefits of aerobic and resistance exercise in subjects affected by Peripheral Arterial Disease (PAD) are scarcely examined in interaction. This study aimed to identify the effects of combined aerobic and resistance exercise programs on walking performance compared with isolated aerobic exercise or with the usual care in patients with intermittent claudication.

**Methods:** A systematic review was conducted following the PRISMA statement. A total of five electronic databases were searched (until October 2019) for randomized and non-randomized controlled trials. The focus comprised PAD patients with intermittent claudication who performed a combined aerobic and resistance exercise program that assessed the walking performance.

**Results:** Seven studies include combined aerobic and resistance exercise vs. isolated aerobic or vs. usual care. The studies represented a sample size of 337 participants. The follow-up ranged from 4 to 12 weeks, 2 to 5 times-per-week. The risk of bias in the trials was a deemed moderate-to-high risk. After the interventions, the percent change in walking performance outcomes had a large variation. In the combined and isolated aerobic programs, the walking performance always improved, while in the usual care group oscillates between the deterioration and the improvement in all outcomes. Combined exercise and isolated aerobic exercise improved the claudication onset distance from 11 to 396%, and 30 to 422%, the absolute claudication distance from 81 to 197%, and 53 to 121%, and the maximal walking distance around 23 and 10%, respectively.

**Conclusions:** Currently, there is insufficient evidence about the effects of combined aerobic and resistance exercise compared to isolated aerobic exercise or usual care on walking performance. However, despite the low quality of evidence, the combined aerobic and resistance exercise seems to be an effective strategy to improve walking performance in patients with intermittent claudication. These combined exercise modes or isolated aerobic exercise produce positive and significant results on walking performance. The usual care approach has a trend to deteriorate the walking performance. Thus, given the scarcity of data, new randomized controlled trial studies that include assessments of cardiovascular risk factors are urgently required to better determine the effect of this exercise combination.

## Introduction

Peripheral arterial disease (PAD) is an occlusive atherosclerotic disease that affects blood vessels, reducing the blood flow of the lower limbs (Vascular Disease Foundation, 2012). The most common symptom is intermittent claudication (IC) affecting millions of people around the world (Gardner et al., 2014). The discomfort associated with IC contributes to a further sedentary lifestyle, that decreases physical fitness, and aggravates cardiovascular risk factors (Winzer et al., 2018). Among patients with IC, a shorter distance and a lower speed walking were associated with an increased risk of cardiovascular and all-cause of mortality (Morris et al., 2014; McDermott et al., 2016), which confirms the importance of an adequate level of physical fitness in this population (Leeper et al., 2013). Indeed, as PAD severity increases, the walking performance progressively decreases (Silva Rde et al., 2015).

Thereby, the promotion of exercise training in patients with PAD and IC is an important non-pharmacological strategy to treat and prevent this disease, which provides favorable systemic vascular effects that may reduce cardiovascular events and improve blood perfusion in PAD (McDermott et al., 2009). Supervised exercise programs have a higher efficacy in improving the physical fitness in particular the walking performance (i.e., claudication onset distance [COD], absolute claudication distance [ACD] or maximal walking distance [MWD]) and the symptomatology of this disease when compared to unsupervised exercise programs (McDermott et al., 2009; Mays and Regensteiner, 2013; McDermott, 2013). However, a recent meta-analysis found that structured home-based exercise programs are effective at improving walking performance in patients with PAD (Golledge et al., 2019). According to guidelines on the diagnosis and treatment of PAD by American Heart Association/American College of Cardiology (AHA/ACC) and European Society of Cardiology/European Society for Vascular Surgery (ESC/ESVS), patients should realize a supervised exercise walking program for a minimum of 30/45 min per session, performed at least 3 times per week for a minimum of 12 weeks (Gerhard-Herman et al., 2017; Aboyans et al., 2018). However, to reduce cardiovascular risk factors and avoid premature mortality, older adults should perform aerobic exercise and also do muscle-strengthening exercises (Piercy et al., 2018; Kraus et al., 2019). A meta-analysis shows that resistance training clinically improved treadmill and flat ground walking performance in patients with PAD (Parmenter et al., 2019). However, in the available literature, the benefits of aerobic and resistance exercises in peripheral arterial disease are commonly studied alone. The combination of these two modes is more effective in the chronic modification of cardiovascular risk factors in healthy elderly men (Sousa et al., 2013), improving quality of life and lipid profiles in patients with coronary disease (Currie et al., 2015) and improving central arterial stiffness and cardiac function in patients with cardiovascular disease (Zhang et al., 2018).

Therefore, there is a clear lack of evidence about the effects of the combined exercise programs to prevent IC progression and PAD treatment. The purpose of this study was to compare the effects of combined aerobic and resistance exercise with another mode of exercise or with the usual care approach on walking performance outcomes.

## Methods

### Eligibility Criteria

This review included randomized controlled trials and non-randomized controlled trials that met the following criteria: (i) contained only patients with PAD and IC; (ii) employed a combined aerobic and resistance exercise program for the treatment of IC compared to another mode of exercise program or to the usual care approach (a non-exercising control group was deemed not essential for these analyses); and (iii) contained measurements of walking performance, such as, COD, ACD, and MWD, assessed with a treadmill protocol or with the 6 min walking test (6 MWT).

The exclusion criteria for the trials were: (i) the treadmill-walking and usual care groups were prescribed medications or surgical interventions for treatment of symptoms of PAD, and the exercise/treatment group was not; (ii) only acute exercise studies (or single exercise sessions); (iii) review articles; (iv) systematic reviews; and (v) case reports.

### Information Sources

Due to the lower overall sample size and the heterogeneity of protocols used, a systematic review was conducted without meta-analysis. Studies were identified by searching electronic databases and hand-searched reference lists of articles, to evaluate the effects of combined aerobic and resistance exercise programs and isolated aerobic exercise or usual care, on walking performance in patients with PAD and IC. Searches were limited to the English language, and no limits were applied to publication dates. This search was applied to Medline (Pubmed), Web of Science, B-On, Cochrane Central Register of Controlled Trials (CENTRAL), and LILACS. The last search was run on 30 October 2019.

### Search Strategy

The following keywords and Boolean operators to search all trials registers and databases were used: (((“peripheral arterial disease”) OR (“peripheral artery disease”) OR (“arterial occlusive disease”) OR (“arterial occlusive diseases”) OR (“arterial obstructive disease”) OR (“peripheral vascular disease”) OR (“peripheral angiopathy”) OR (“claudication”) OR (“claudicant”) OR (“claudicants”)) AND ((“aerobic and resistance”) OR (“aerobic and strength”) OR (“aerobic and strengthening”) OR (“circuit exercise”) OR (“circuit exercises”) OR (“circuit program^*^”) OR (“circuit training”) OR (“circuit trainings”) OR (“circuit-based”) OR (“combined aerobics”) OR (“combined endurance”) OR (“combined endurances”) OR (“combined exercise”) OR (“combined exercises”) OR (“combined muscle resistance”) OR (“combined muscle strength”) OR (“combined muscle strengthening”) OR (“combined program^*^”) OR (“combined resistance”) OR (“combined resistances”) OR (“combined strength”) OR (“combined strengthening”) OR (“combined strengths”) OR (“combined training”) OR (“combined walk”) OR (“combined walking”) OR (“combined weight bearing”) OR (“combined weight-lifting”) OR (“concurrent aerobics”) OR (“concurrent endurance”) OR (“concurrent endurances”) OR (“concurrent exercise”) OR (“concurrent exercises”) OR (“concurrent muscle resistance”) OR (“concurrent muscle strength”) OR (“concurrent muscle strengthening”) OR (“concurrent program^*^”) OR (“concurrent resistance”) OR (“concurrent resistances”) OR (“concurrent strength”) OR (“concurrent strengthening”) OR (“concurrent training”) OR (“concurrent walk”) OR (“concurrent walking”) OR (“concurrent weight bearing”) OR (“concurrent weight-lifting”) OR (“walking and resistance”) OR (“walking and strength”))).

### Study Selection

Eligibility assessment was performed independently in an unblinded standardized manner by two reviewers (IM and CA). Disagreements between reviewers were resolved by consensus. All the work of revision was oriented according to the Preferred Reporting Items for Systematic reviews and Meta-Analyses (PRISMA) protocol (Liberati et al., 2009; Moher et al., 2009). For the PRISMA checklist see Supplementary Material.

### Data Collection Process

One review author extracted the data from the included studies, and the second author checked the extracted data. Disagreements were resolved by discussion between the two review authors; if no agreement could be reached, it was planned a third author would decide (NS). We contacted two authors for further information, but just one answered and provided numerical data.

### Data Items

The extracted outcomes data will be analyzed through a narrative synthesis. Information was extracted from each included trial on: (i) characteristics of participants (including age, gender, and stage of disease); (ii) intervention (including type, intensity, duration and week frequency of exercise) of the combined exercise program vs. the isolated aerobic exercise or vs. the usual care approach (encouraged to follow the exercise recommendations at home); (iii) walking protocol (treadmill graded protocol, treadmill constant protocol, and 6 MWT; and (iv) outcome measures (COD, ACD, MWD, and ABI).

The outcome results were reported as mean ± SD and were considered significant when *p* < 0.05 from the study statistical analyses. Since the treadmill-walking test was performed at a constant speed, there is a direct relationship between time and walking distance. To compare the results of the Hiatt et al. (1994) study, the data of claudication onset time and absolute claudication time originally presented in minutes was converted to seconds. The data related to COD and ACD were calculated since a constant-load exercise protocol was performed. The calculated distance results were considered statistically significant if the original results were also significant. Regarding the study by Tebbutt et al. (2011), the results were originally presented as a median, but for comparison purposes with the other studies, the COD and ACD mean values were estimated, after approval by the author. In the studies of Jakubseviciene et al. (2014) and Kropielnicka et al. (2018), since the data were presented in chart forms, the authors were contacted to provide the exact numerical data, to be able to compare with the other studies, however, they did not answer.

### Quality Assessment

The methodological quality of each selected study (*n* = 7) used was the Physiotherapy Evidence Database (PEDro) rating scale. In the PEDro scale, points are only awarded when a quality criterion is clearly satisfied. Any disputes were resolved by consensus, or by a third author (NS) when necessary.

## Results

### Study Selection

**Figure 1:**
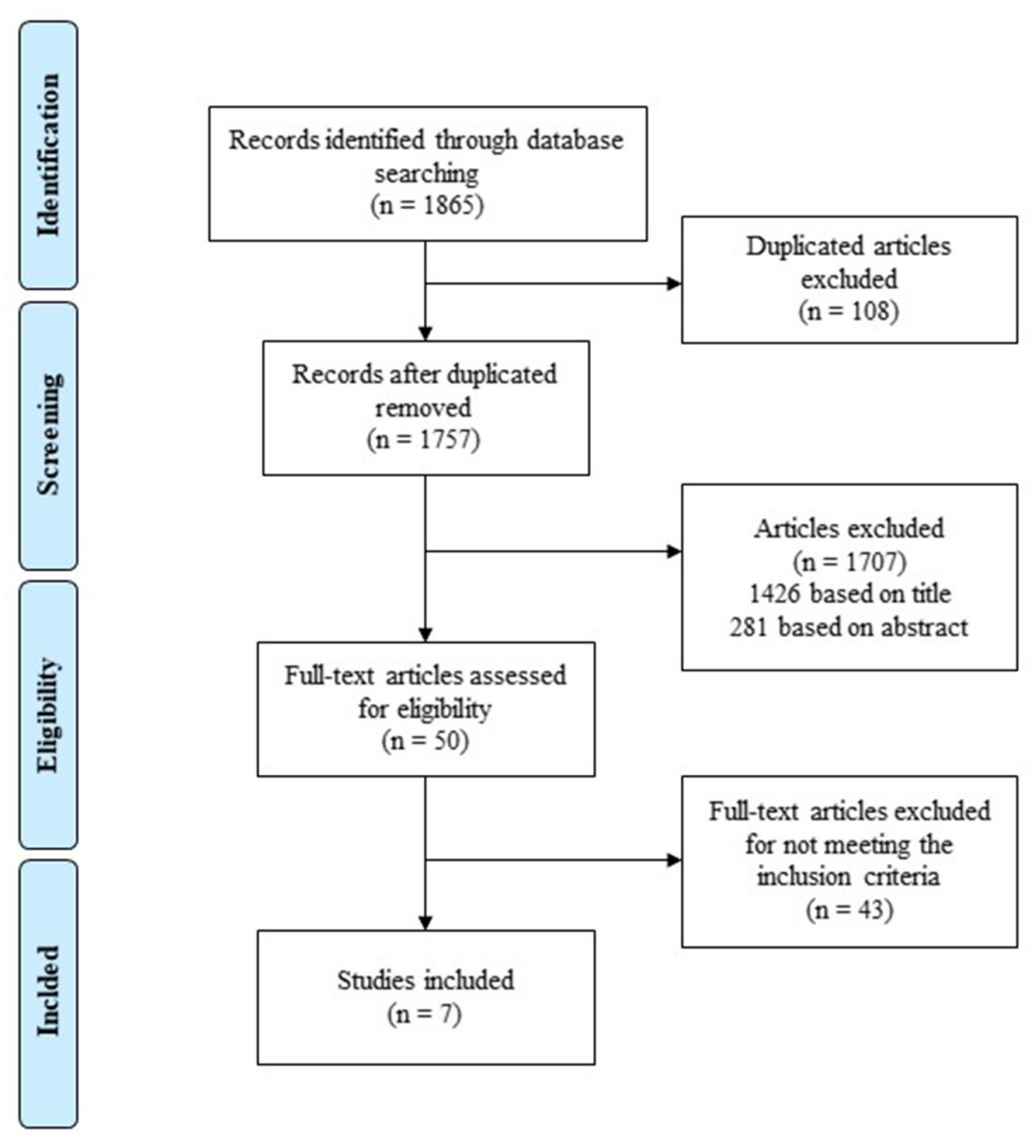
Flow diagram of the literature serch according to items in the PRISMA guidelines.

A total of 1,865 potentially relevant articles were identified through the database search, and a total of 108 studies were discarded because were duplicated. A total of 1,426 articles were removed after reading titles and 281 after reviewing the abstracts since they clearly did not meet the inclusion criteria. The full text of the remaining 50 references was examined in detail, and 43 studies did not meet the inclusion criteria. Seven studies met the inclusion criteria and were included in this systematic review (Figure 1). No unpublished relevant studies were obtained.

A summary of eligible studies is shown in Table 1. The studies were implemented in four continents: four from Europe (Mosti et al., 2011; Tebbutt et al., 2011; Jakubseviciene et al., 2014; Kropielnicka et al., 2018), one from America (Hiatt et al., 1994), one from Africa (Parr et al., 2009), and one from Oceania (Delaney et al., 2014). One trial was not randomized (Mosti et al., 2011), and one used a pseudo-randomization method (Kropielnicka et al., 2018).

**Table 1:** Summary of eligible studies.

**Study/Participants**	**Pathology**	**Primary outcome**	**Intervention vs. comparative**	**Duration/Frequency**	**Protocol**	**Training description/progression**	**Main Results**
**COMBINED AEROBIC AND RESISTANCE EXERCISE VS. ISOLATED AEROBIC EXERCISE**							
^a^Hiatt et al. (1994) *N* = 20 (20 males) CB = 10 (82 ± 17 years) AER = 10 (70 ± 12 years)	PAD ABI < 0.94 IC	COD ACD	Treadmill + Resistance Training vs. Treadmill	12 weeks 3×	**GRAD** 3.2Km/h 0% grade: ↑3,5% every 3 min **CONST** 3.2Km/h % grade: where begin CI on Graded	**CB:** 50′ treadmill walking with work-rest-cycle (claudication after 3–5 min of exercise) **AND** 30′ of 3 Exercises, 3 sets, 6 reps (cuff weight) **AER:** 60′ Treadmill walking with work-rest-cycle (claudication after 3–5 min of exercise) (↑ grade and speed weekly)	**COD** **Baseline and after 12 weeks with a GRAD** **CB:** 165 ± 69 and 272 ± 208 m; +65 %Change **AER:** 176 ± 107 and 357 ± 229 m *(p < 0.05);* +103 %Change **Baseline and after 12 weeks with a CONST** **CB:** 133 ± 59 and 661 ± 1,248 m; +396 %Change **AER:** 187 ± 107 and 976 ± 1,211 m *(p < 0.05);* +422 %Change **ACD** **Baseline and after 12 weeks with a GRAD** **CB:** 389 ± 144 and 704 ± 352 m *(p < 0.05)* +81 %Change **AER:** 512 ± 304 and 784 ± 389 m *(p < 0.05);* +53 %Change **Baseline and after 12 weeks with a CONST** **CB:** 400 ± 288 and 1,189 ± 773 m *(p < 0.05)*; +197 %Change **AER:** 704 ± 581 m and 1,627 ± 955 m *(p < 0.05)*; +131 %Change
Delaney et al. (2014) *N* = 35 (26 Males) CB = 17 (69 ± 10 years) AER = 18 (73 ± 9 years)	ABI < 0.90 IC	COD MWD	Treadmill + Resistance Training vs. Treadmill	12 weeks 2×	**6 MWT**	**CB:** 5 exercises, 3 sets, 8–12 reps hamstring curls, seated calf press, leg press, knee extension, and hip abduction/adduction with minimum level of resistance. ↑ 5% resistance when achieve 12 reps/set **AND** walk on treadmill until the onset CI. Rest until pain solved. Repeat for the duration of session. **AER:** 60′ treadmill work-rest-cycle; Initial speed determined by distance in 6MWT. ↑ 10%grade when walking 10min without IC.	**COD** **Baseline and after 12 weeks with 6MWT** **CB:** 170 ± 90 and 188 ± 127 m; +11 %Change **AER:** 170 ± 82 and 221 ± 97 m *(p ≤ 0.03);* +30 %Change **MWD** **Baseline and after 12 weeks with 6MWT** **CB:** 358 ± 68 and 439 ± 188 m; +23 %Change **AER:** 371 ± 94 and 409 ± 70 m *(p ≤ 0.01)*; +10 %Change
Jakubseviciene et al. (2014) *N* = 145 CB = 72 AE = 73	PAD Stage II–III **Fontaine** IC	COD MWD ABI	Treadmill, ergometers, steppers, stair climbing, jogging + Resistance training vs. Track walking, stair climbing, treadmill	4 weeks 4/5×	**6MWT**	**CB:** 60′ session; 10′ warm-up, 40′ of treadmills, ergometers, steppers, stair climbing, jogging + resistance devices (maximum heart rate between 60 and 85%) and 10′ cool-down. **AER:** 45′ session; 5–10′ warm-up (dynamic breathing and stretching exercises), 25–35′ treadmill exercise, track walking, stair climbing, and 5–10′ cool-down (static and dynamic breathing and stretching exercises)	**COD** (data not shown) **Baseline and after 4 weeks with a 6MWT** **CB:** *(p < 0.05)* **AER:** *(p < 0.05)* **MWD** (data not shown) **Baseline and after 4 weeks with a 6MWT** **CB:** *(p < 0.05)*; **AER:** *(p < 0.05)*; **ABI** (data not shown) **Baseline and after 4 weeks with a 6MWT** **CB**: *(N.S.)*; **AER:** *(N.S.)*; After 4 weeks there was no differences between groups.
Kropielnicka et al. (2018) *N* = 59 CB *N* = 28 (68 ± 9 years) AER *N* = 31 (67 ± 7 years)	PAD Stage II **Fontaine** ABI < 0.90 IC	COD ACD MWD	Nordic walking + Resistance Training vs. Treadmill	12 weeks 3×	**GRAD** 3.2 Km/h 0% grade: ↑2% every 2 min **6MWT**	**CB:** 60′ session; 10′ warm-up with the poles, 45′ of Nordic walking (work-rest-cycle) 5′ of stretching and breathing exercises **AND** 70–80% of the maximum (plantar and dorsal flexors of the ankle joint, or in patients with high arterial occlusion, training for flexors and extensors of the knee joint in both limbs was introduced). One exercise, 10 reps, angular velocity: 60, 120, 180, 240, and 300 and 300, 240, 180, 120, and 60% (according to the pyramid rule). 100 movements per limb. One minute rested after each velocity. **AER:** 45′ session; Constant treadmill walking (3.2 km/h, 12% grade) work-rest-cycle	**COD** (data not shown) **Baseline and after 12 weeks with a GRAD** **CB:** *(N.S.)* **CP:** *(p < 0.05)* **ACD** (data not shown) **Baseline and after 12 weeks with a GRAD** **CB:** *(p < 0.05)*; **CP:** *(p < 0.05)*; **MWD** (data not shown) **Baseline and after 12 weeks with a 6MWT** **CB**: *(p < 0.05)*; **CP:** *(p < 0.05)*; CB vs. CP *(NS)*
**COMBINED AEROBIC AND RESISTANCE EXERCISE VS. USUAL CARE**							
Parr et al. (2009) *N* = 16 (10 males) CB = 8 (57 ± 14 years) UC = 8 (62 ± 10 years)	PAD IC	COD ACD MWD ABI	Treadmill + 1. Resistance Training, 2. Floor exercises, 3. Spin class vs. Usual care	6 weeks 3×	**GRAD** 3.2 Km/h 0% grade: ↑2% every 2 min **6MWT**	**CB:** 45′ session; 10–20′ Treadmill with Work-rest-cycle *↑ weekly by increasing the walking speed (by 0.3 km/h) or the gradient (by 1%)* **AND** +1 × /week 5′ cycled and 15′ resistance training (upper and lower body) 6 exercises; 15 reps floor exercises +1 × /week Floor exercises (step, aerobic or core stabilization); + 1x/week 15′ spin class And 5′ of stretching at the end. **UC:** Usual care (were advised to walk as much as possible at home)	**COD** **Baseline and after 6 weeks with a GRAD** **CB:** 125 ± 167 and 255 ± 201 m; +104 %Change **UC:** 175 ± 123 and 175 ± 136 m; 0 %Change **Baseline and after 6 weeks with a 6MWT** **CB:** 121 ± 108 and 152 ± 109 m; +26 %Change **UC:** 192 ± 147 and 116 ± 75 m; −40 %Change After 6 weeks, CB improve COD compared to UC *(p < 0.05)*. **ACD** **Baseline and after 6 weeks with a GRAD** **CB:** 290 ± 210 and 485 ± 224 m; +67 %Change **UC:** 460 ± 200 and 430 ± 151 m; −7 %Change After 6 weeks, CB improve ACD compared to UC *(p < 0.05)*. **MWD** **Baseline and after 6 weeks with a 6MWT** **CB:** 368 ± 124 and 421 ± 128 m; +14 %Change **UC**: 415 ± 144 and 408 ± 125 m; −2 %Change **ABI:** Data not shown (NS)
Mosti et al. (2011) *N* = 20 (15 Males) CB = 10 (65 ± 5 years) UC = 10 (66 ± 5 years)	PAD Stage II **Fontaine** ABI ≤ 0.90 IC	ACD	Plantar flexion + Resistance Training vs. Usual care	8 weeks 3×	**GRAD** 3.2 Km/h 0% grade: ↑3,5% every 3 min	**CB:** 60′ session; 4-min work intervals (one leg each time) Initial workload: 80% of maximal workload and flexion frequency 55–65 rpm **AND** horizontal dynamic leg, 4 sets, 5 reps (initial work load at 85–90% 1RM) ↑5 kg when they are able to complete all sets and reps. **UC:** Usual care (received exercise guidelines in accordance to recommendations to PAD).	**ACD** **Baseline and after 8 weeks with a GRAD** **CB:** 1,099 ± 463 and 1,203 ± 451 m *(p < 0.01);* +10 %Change **UC:** 936 ± 353 and 928 ± 347 m; −1%Change After 8 weeks, CB improve ACD compared to UC (*p < 0.01)*
Tebbutt et al. (2011) *N* = 42 (28 males) CB = 24 (66 years) UC = 18 (71 years)	PAD Stage II **Fontaine** ABI < 0.90 IC	COD ACD ABI	Walk + Plantar flexion vs. Usual care	12 weeks 3×	**CONST** 3.2 Km/h 10% grade	**CB:** (unsupervised) Participants were advised to walk **AND** Step it Pedal (resistance of pedal ≈6 kg) 2′ exercise/2′ rest, ×10 times (20 min of resistance exercise and 40′ in total). **UC:** Usual care (Participants were advised to walk).	**COD** **Baseline and after 12 weeks with a CONST** **CB:** 77 ± 55 and 115 ± 96 m; +49 %Change **UC**: 113 ±107 and 140 ±116 m; +24 %Change **ACD** **Baseline and after 12 weeks with a CONST** **CB:** 173 ± 103 and 201 ± 109 m; +16 %Change **UC:** 237 ± 174 and 277 ± 200 m; +17 %Change **ABI** Data not shown *(NS)*

*CB, Combined aerobic and resistance exercise group; AER, Aerobic exercise; UC, Usual care approach group; GRAD, graded protocol; CONST, constant protocol; 6MWT, 6 min walking test; COD, claudication onset distance/time; ACD, absolute claudication distance/time; MWD, maximal walking distance; NS, Not significant*.

a *For the CB group, data from the second 12 weeks of combined training were considered since in the first 12 weeks the group was a control group. For AER were considered the data of the first 12 weeks of treadmill-walking program*.

### Participants

The total number of 337 participants were involved in the seven studies, 99 were men, 34 were female, and 204 were not reported. All patients had IC and ABI at rest ≤0.90 (except Hiatt et al., 1994 that have an ABI < 0.94 and Jakubseviciene et al., 2014 that didn't report). A total of 337 participants, 169 of whom were allocated to the combined exercise group, 132 to the isolated aerobic exercise group, and 36 to the usual care group; 43 among them were excluded from statistical analysis across all studies. The mean age among participants ranged from 57 to 82 years, and the mean body mass index, when reported, was 28.0 ± 4.3 kg/m^2^.

In the present systematic review, the majority of patients with PAD reported cardiovascular risk factors and other comorbidities conditions, at least 18% were current smokers, 15% of the patients had hypertension, 12% had hyperlipidemia, 7% had diabetes, 3% had coronary heart disease, 2% had a history of cerebrovascular disease, and 8% had other diseases (Table 2). These values are probably higher since we only had access to 45% of the sample characterization.

**Table 2:** Cohort characteristics.

	**Hiatt et al. (1994)**	**Parr et al. (2009)**	**Mosti et al. (2011)**	**Tebbutt et al. (2011)**	**Delaney et al. (2014)**	**Jakubseviciene et al. (2014)**	**Kropielnicka et al. (2018)**	**Total (*N*)**	**Total (%)**
*N*	20	16	20	42	35	145	59	337	100
Males (%)	100%	63%	75%	67%	74%	NR	NR	99	29
Females (%)	0%	37%	25%	32%	26%	NR	NR	34	10
Age^*^ (years ± SD)	CB: 82 ± 17 AER: 70 ± 12	CB: 57 ± 14 UC: 62 ± 10	CB: 65 ± 5 UC: 66 ± 5	CB: 66 UC: 71	CB: 69 ± 10 AER: 74 ± 9	NR	CB: 68 ± 9 AER: 67 ± 7	–	–
Current smoker (%)	95%	NR	25%	29%	74%	NR	NR	62	18
Hipertension (%)	70%	NR	45%	NR	83%	NR	NR	52	15
Hyperlipidemia (%)	35%	NR	NR	NR	92%	NR	NR	39	12
Diabetes (%)	NR	NR	20%	5%	46%	NR	NR	22	7
Coronary heart disease (%)	45%	NR	NR	NR	NR	NR	NR	9	3
Cerebrovascular disease (%)	30%	NR	NR	NR	NR	NR	NR	6	2
Others (%)	20%	NR	40%	NR	40%	NR	NR	26	8

* *Values are mean ± SD; CB, Combined aerobic and resistance exercise group; AER, aerobic exercise group; UC, Usual care group; NR, not reported*.

The combined aerobic and resistance exercise group was compared with the isolated aerobic exercise group in 4 studies and with the usual care approach in three studies.

### Intervention

All trials have used combined aerobic and resistance exercise, however, there are some differences between the combined exercise programs analyzed. The most common aerobic exercise mode used was treadmill-walking exercise, however, one study used a plantar flexion ergometer (Mosti et al., 2011), one study used a nordic-walking training (Kropielnicka et al., 2018), and another one used more than one mode of aerobic exercise including treadmill-walking exercise. Interval walking to moderate-maximum claudication pain was the most common prescription. The work-rest-cycle (i.e., exercise until a specific pain level, usually moderate or near maximal, followed by rest until the pain disappears, and repeat the cycle) was used due to claudication, and exercise duration raged from 20 to 50 min. The follow-up ranged from 4 to 12 weeks, and the supervised training frequency ranged from 2 to 5 days per week.

For resistance exercise mode, four studies used plate loaded machines (Parr et al., 2009; Mosti et al., 2011; Delaney et al., 2014; Jakubseviciene et al., 2014), one study used a resistance pedal (Tebbutt et al., 2011), one study used a functional dynamometer under constant angular velocity (Kropielnicka et al., 2018), and another study used the body weight and an additional load resistance with a cuff weight (Hiatt et al., 1994). One study performed upper and lower body exercises, while the majority performed only lower body exercises. The number of exercises varied between 1 and 6, the sets between 1 and 4 [except Kropielnicka et al. (2018) that used 10 sets] and between 5 and 15 repetitions. Except for one study, the combined aerobic and resistance exercise program was home-based and was not supervised (Tebbutt et al., 2011).

### Outcomes

All studies evaluate walking performance, and some used more than one protocol. A total of five studies used a treadmill-walking protocol (Hiatt et al., 1994; Parr et al., 2009; Mosti et al., 2011; Tebbutt et al., 2011; Kropielnicka et al., 2018) and four studies used the 6 MWT (Parr et al., 2009; Delaney et al., 2014; Jakubseviciene et al., 2014; Kropielnicka et al., 2018). In treadmill walking-protocol, two studies used a constant protocol (Hiatt et al., 1994; Tebbutt et al., 2011) and four used a graded protocol (Hiatt et al., 1994; Parr et al., 2009; Mosti et al., 2011; Kropielnicka et al., 2018).

The COD was defined as the distance on the moment in which claudication pain starts; ACD was defined as the distance that had been walked until patients were unable to continue due to the increased intensity of claudication pain, and; MWD was defined as the total walking distance reached at the end of the protocol (work-rest-cycle sum). For example, a patient walked 270 m without pain (COD) and had to stop 950 m after starting due to IC (ACD). After the pain released, he resumed his walk, and at the end of the 30-min session had walked a total of 1,600 m (MWD).

### Studies Quality Assessment

The following criteria were used to classify the methodological quality: a score from PEDro equal or greater than 7 (*n* = 1) indicates a high methodological quality; scores between 5 and 6 (*n* = 2) indicate a moderate methodological quality; and scores below 5 (*n* = 4) indicated low methodological quality. The highest PEDro rating was 7, with an average of 5 among the included studies (Table 3).

**Table 3:** Score of articles according to PEDro scale score.

	**Hiatt et al. (1994)**	**Parr et al. (2009)**	**Mosti et al. (2011)**	**Tebbutt et al. (2011)**	**Delaney et al. (2014)**	**Jakubseviciene et al. (2014)**	**Kropielnicka et al. (2018)**
1. Study eligibility criteria specified	0	0	0	1	1	1	1
2. Random allocation	1	1	0	1	1	1	1
3. Concealed allocation	0	0	0	0	0	0	0
4. A measure of similarity between groups at baseline	1	1	1	1	1	1	1
5. Subject blinding	0	0	0	0	0	0	0
6. Therapist blinding	0	0	0	0	0	0	0
7. Assessor blinding	0	0	0	0	1	0	0
8. Less than 15% dropouts	1	0	1	0	1	1	0
9. Intention to treat analysis	0	0	0	0	1	0	0
10. Between-group statistical comparative	1	1	1	1	1	1	1
11. Point measures and validity data	1	1	1	1	1	1	1
Total points	5	4	4	4	7	5	4

### Combined Exercise vs. Aerobic Exercise

Statistical significant improvements were found on COD in the combined exercise group after 4 weeks (Jakubseviciene et al., 2014) and in the isolated aerobic group after 12 weeks (Hiatt et al., 1994; Delaney et al., 2014; Kropielnicka et al., 2018). Between groups, there were no significant differences. The combined exercise group showed a trend to improve the COD between 11 and 396% (+18 and +528 m), while the isolated aerobic group showed a trend to improve between 30 and 422% (+51 and +789 m) (Table 4).

**Table 4:** Results of the combined aerobic and resistance exercise vs. isolated treadmill-walking or usual care approach in walking performance.

	**Study**	**Duration**	**Protocol**	**Intervention**	**Baseline (m)**	**Post-training (m)**	**M ≠ in group**	**%Change group**
**COD**								
CB vs. AER	Hiatt et al. (1994)	12 weeks	Grad	CB: Treadmill + Resistance	165 ± 69	272 ± 208	107	65
				AER: Treadmill	176 ± 107	357 ± 229^*^	181	103
	Hiatt et al. (1994)	12 weeks	Const	CB: Treadmill + Resistance	133 ± 59	661 ± 1,248	528	396
				AER: Treadmill	187 ± 107	976 ± 1,211^*^	789	422
	Delaney et al. (2014)	12 weeks	6 mwt	CB: Treadmill + Resistance	170 ± 90	188 ± 127	18	11
				AER: Treadmill	170 ± 82	221 ± 97^**^	51	30
	Jakubseviciene et al. (2014)	4 weeks	6 mwt	CB: Treadmill, ergometers, steppers + Resistance	NR	NR^*^	–	–
				AER: Track walking, stair climbing, Treadmill	NR	NR^*^	–	–
	Kropielnicka et al. (2018)	12 weeks	Grad	CB: Nordic Walk + Resistance	NR	NR	–	–
				AER: Treadmill	NR	NR^*^	–	**–**
CB vs. UC	Parr et al. (2009)	6 weeks	Grad	CB: Treadmill + Resistance	125 ± 167	255 ± 201	130	104
				UC: Usual Care	175 ± 123	175 ± 136	0	0
	Parr et al. (2009)	6 weeks	6 mwt	CB: Treadmill + Resistance	121 ± 108	152 ± 109^°^	31	26
				UC: Usual Care	192 ± 147	116 ± 75	−76	−40
	Tebbutt et al. (2011)	12 weeks	Const	CB: Walk + Plantar Flex Unsupervised	77 ± 55	115 ± 96	38	49
				UC: Usual Care	113 ± 107	140 ± 116	27	24
**ACD**								
CB vs. AER	Hiatt et al. (1994)	12 weeks	Grad	CB: Treadmill + Resistance	389 ± 144	704 ± 352^*^	315	81
				AER: Treadmill	512 ± 304	784 ± 389^*^	272	53
	Hiatt et al. (1994)	12 weeks	Const	CB: Treadmill + Resistance	400 ± 288	1,189 ± 773^*^	789	197
				AER: Treadmill	704 ± 581	1,627 ± 955^*^	923	131
	Kropielnicka et al. (2018)	12 weeks	Grad	CB: Nordic Walk + Resistance	NR	NR^*^	–	–
				AER: Treadmill	NR	NR^*^	–	–
CB vs. UC	Parr et al. (2009)	6 weeks	Grad	CB: Treadmill + Resistance	290 ± 210	485 ± 224^°^	195	67
				UC: Usual Care	460 ± 200	430 ± 151	−30	−7
	Mosti et al. (2011)	8 weeks	Grad	CB: Plantar Flex + Strength	1,099 ± 463	1,203 ± 451^***^, ^°°^	104	10
				UC: Usual Care	936 ± 353	928 ± 347	−8	−1
	Tebbutt et al. (2011)	12 weeks	Const	CB: Walk + Plantar Flex Unsupervised	173 ± 103	201 ± 109	28	16
				UC: Usual Care	237 ± 174	277 ± 200	40	17
**MWD**								
CB vs. AER	Delaney et al. (2014)	12 weeks	6 mwt	CB: Treadmill + Resistance	358 ± 68	439 ± 188	81	23
				AER: Treadmill	371 ± 94	409 ± 70^***^	38	10
	Jakubseviciene et al. (2014)	4 weeks	6 mwt	CB: Treadmill, ergometers, steppers + Resistance	NR	NR^*^	–	–
				AER: Track walking, stair climbing, Treadmill	NR	NR^*^	–	–
	Kropielnicka et al. (2018)	12 weeks	6 mwt	CB: Nordic Walk + Resistance	NR	NR^*^	–	–
				AER: Treadmill	NR	NR^*^	–	–
CB vs. UC	Parr et al. (2009)	6 weeks	6 mwt	CB: Treadmill + Resistance	368 ± 124	421 ± 128	53	14
				UC: Usual Care	415 ± 144	408 ± 125	−7	−2

CB, Combined aerobic and resistance exercise group; AER, Aerobic exercise group; UC, Usual care approach group; Grad, Graded protocol; Const, Constant protocol; 6 mwt, 6 min walking test; %change, Magnitude of percent change (%Change = (post-training – baseline)/baseline*100).

* *Different from pre-training within group, p < 0.05*.

** *Different from pre-training within group p < 0.03*.

*** *Different from pre-training within group, p ≤ 0.01*.

° *Difference between groups, p < 0.05*.

°° *Difference between groups, p ≤ 0.01*.

Statistical significant improvements were found on ACD in the combined exercise and isolated aerobic exercise group after 12 weeks (Hiatt et al., 1994; Kropielnicka et al., 2018). Between groups, there were no significant differences. The combined exercise group showed a trend to improve the ACD between 81 and 197% (+315 and +789 m) while the isolated aerobic exercise group showed a trend to improve between 53 and 131% (+272 and +923 m) (Table 4).

Statistical significant improvements were found in MWD in the combined group after 4 and 12 weeks (Jakubseviciene et al., 2014; Kropielnicka et al., 2018) and in the isolated aerobic group after 12 weeks (Delaney et al., 2014; Kropielnicka et al., 2018). There were no significant differences between groups. The combined exercise group showed a trend to improve the MWD around 23% (+81 m), while the isolated aerobic exercise group showed a trend to improve around 10% (+38 m) (Table 4).

### Combined Exercise vs. Usual Care Approach

Statistical significant improvements were not found in COD after 6 or 12 weeks of combined exercise and usual care approach. However, between groups, statistical differences were found in COD with improvements found only in the combined group (Parr et al., 2009). The combined exercise group showed a trend to improve the COD between 26 and 104% (+31 and +130 m), while the usual care deteriorated or improved between −40 and 24% (−76 and +27 m) (Table 4).

Statistical significant improvements were found on ACD only in the combined exercise group after 8 weeks (Mosti et al., 2011). Improvement in the combined group after 6 weeks (Parr et al., 2009) and 8 weeks (Mosti et al., 2011) compared to usual care were found in ACD, with statistical differences. The combined exercise group showed a trend to improve the ACD between 10 and 67% (+104 and +195 m), while usual care deteriorated or improved between −7 and 17% (−30 and +40 m) (Table 4).

There were no significant improvements in MWD, along with the intervention. However, the combined exercise group showed a slight trend to improve the MWD around 14% (+53 m) while the usual care group deteriorated −2 (−7 m) (Table 4).

These walking performance results were quite variable in all groups, and their magnitude change could be affected by the applied walking protocol test (Table 4).

Rest ABI was observed in combined exercise, isolated aerobic exercise, and usual care groups; however, no changes were found in this variable (Table 1).

## Discussion

The overall findings of this systematic review suggest that both combined aerobic and resistance and isolated aerobic programs improve claudication onset distance, absolute claudication distance, and maximal walking distance, with no significant differences found between these strategies. However, the combined aerobic and resistance exercise programs improved claudication onset distance and absolute claudication distance when compared to the usual care approach.

### Combined Exercise vs. Aerobic Exercise

The positive effects of the exercise programs in IC patients are often related to improved endothelial function, skeletal muscle metabolism, blood viscosity, pain tolerance, and inflammatory responses (Stewart et al., 2002). These mechanisms are linked to exercise benefits in PAD and can counteract the disease progression. The increase in the majority of the walking performance outcomes suggest that the key to induce improvements is a well-structured exercise program, with isolated aerobic exercise or combined with resistance exercise, that complies with the specific PAD exercise doses. Accordingly, home-based exercise programs, if structured, involving adequate supervision and aimed to motivate the patients to increase physical activity levels or other behavioral interventions, can also improve walking performance outcomes (Golledge et al., 2019). The walking performance improvements are associated with significant enhancements in functional status, quality of life (Lauret et al., 2014; Novakovic et al., 2017), and also to considerable decreases in sedentary levels that may help in the management of cardiovascular risk factors (Morris et al., 2014; McDermott et al., 2016; Novakovic et al., 2017).

Although there are no significant differences in COD, ACD, and MWD between the exercise groups, it is consensual that PAD patients have lower limb muscle strength and less calf muscle mass related to the general population, which causes functional impairment, that isolated aerobic exercise by itself cannot overtake. Isolated resistance training can attenuate age-related changes in muscle function (Papa et al., 2017), and can be associated with improvements in balance (Gonzalez et al., 2014), muscle strength, walking performance (Ritti-Dias et al., 2010; Wang et al., 2010), psychological well-being, and quality of life (Pedersen et al., 2017). However, among patients with an increased risk of cardiovascular disease, the combined training may provide more cardiovascular benefits, when compared to time-matched aerobic or resistance training alone (Schroeder et al., 2019). A single session of combined aerobic and resistance exercise improves blood flow and leg vascular resistance similar to a single aerobic session; however, combined exercise promotes better effects on oxidative stress responses (Lima et al., 2018), regarded as morbidity and mortality marks in several populations (Yucel et al., 2015; Zhang et al., 2015). Combined aerobic and resistance exercise may induce improvements in other outcomes that were not specifically studied in this systematic review. In three studies included in this systematic review, the walking performance outcomes improved (Mosti et al., 2011; Delaney et al., 2014; Kropielnicka et al., 2018), and also positive effects were found on lower limb muscle strength parameters, such as, peak torque, total work, and average power in the combined exercise when compared to isolated aerobic exercise or to usual care approach. Combined aerobic and resistance exercise, in addition to improving walking performance, also seems to increase lower limb muscle strength, which may lead to a better quality of life. One advantage of combined exercise is the fact that patients feel it as being less painful than isolated walking exercise (Ritti-Dias et al., 2010), which could help to improve exercise adherence.

In the combined groups of this systematic review, the majority of aerobic exercise sessions consisted of supervised treadmill-walking while the resistance exercise sessions, showed heterogeneity between studies, concerning the number of sets, number of repetitions and number of total exercises. The studied variables are, as well, different from each other, and in one study, the exercise characteristics were not described (Jakubseviciene et al., 2014). The exercise programs duration, ranged from 4 to 12 weeks and the study with the shortest duration (Jakubseviciene et al., 2014) achieve significant improvements in walking performance both in combined and isolated exercise groups, which can be explained because: (i) both groups performed the exercise program with a higher exercise weekly frequency (4/5 times a week), and (ii) both groups performed the exercise program immediately after a lower limb arterial surgery. The total duration of the exercise sessions (in min) and the partial aerobic exercise duration of the combined and isolated aerobic exercise programs are relevant points to note. In fact, one study implemented a total combined session duration of 90 min and matched the same aerobic exercise duration (50 min) in both combined and isolated aerobic exercise (Hiatt et al., 1994). Also, another study promoted a slightly higher total duration (+15 min) in combined exercise (Jakubseviciene et al., 2014), and two studies implemented the same session duration (Delaney et al., 2014; Kropielnicka et al., 2018), promoting a lower training volume of each exercise mode. Thus, it may not meet the current PAD guidelines, nor achieve the minimum stimulus of the exercise duration to accomplish the same benefits of isolated aerobic or resistance exercise when combined (Gerhard-Herman et al., 2017; Aboyans et al., 2018).

Supervised exercise programs not always reveal significant improvements in COD. However, ACD has always improved, which reveals a better capacity to walk long distances. The pain during exercise does not disappear, in fact, it seems that the pain is better tolerated and/or patients perceive a lower pain level. Regarding ABI results, as expected, no changes were found in combined, isolated or usual care approach groups. Exercise does not directly eliminate systemic atherosclerosis but may increase the muscle oxygen delivery or oxygen utilization through microvascular alterations and improvement of endothelial dilatation in collateral blood vessels (Baker et al., 2017).

A wide range of different protocols are used and these present a variety of characteristics, such as, constant and graded load protocols with different speeds, grade, and stage duration, which can promote distinct results, hampering adequate comparison of studies and strategies. The treadmill-walking is the most used protocol to assess the walking performance in PAD patients. When PAD patients are tested in constant protocols, the values of COD and ACD are higher (Hiatt et al., 1994). The differences are also related to the heterogeneity of this population once the same load promotes different patterns of IC. A meta-regression analysis found the highest estimated reliability in COD assessed by constant treadmill protocols, but the graded treadmill protocol promoted the highest reliability in ACD assessments (Nicolai et al., 2009). The MWD can be adequately assessed by the 6MWT and this test better represents walking in daily life compared to treadmill-walking protocols (McDermott et al., 2014; Nordanstig et al., 2014).

### Combined Exercise vs. Usual Care Approach

When combined aerobic and resistance exercise are compared to usual care approaches, there are positive and significant differences in COD and ACD outcomes, except in one study, where the combined exercise program was unsupervised. The unsupervised exercise program combined walking and resistance pedal, and the outcomes presented minor improvements (Tebbutt et al., 2011). This lack of effect can be related to the early interruption of the walking exercise due to intermittent claudication, and also to the inexistence of workload progression in home-based exercises. The usual care approach, have a trend to deteriorate walking performance, considering that PAD is a progressive atherosclerotic disorder that deteriorates over time. These findings are in agreement with several randomized controlled trials and systematic reviews that compared supervised walking exercise to unsupervised exercise and usual care programs (McDermott et al., 2009; Hamburg and Balady, 2011; Vascular Disease Foundation, 2012; Mays and Regensteiner, 2013; McDermott, 2013; Lane et al., 2017).

Two studies with a short program intervention (below 2-months) did not comply with the current PAD exercise guidelines (Gerhard-Herman et al., 2017; Aboyans et al., 2018) but found significant improvements in COD (Parr et al., 2009) and ACD (Parr et al., 2009; Mosti et al., 2011). The study conducted by Parr et al. (2009) promoted an exercise program that consisted to 20 min of treadmill-walking exercise combined with one of the following exercises on different days: (i) cycling for 5 min followed by a 15 min circuit training (15 repetitions of six different upper and lower body exercises) in plate load machines; (ii) floor exercises consisting of either bench-step, aerobics or core stabilization exercises; and (iii) 15 min spin (cycle) class. The study conducted by Mosti et al. (2011) used an interesting modify plantar flexion ergometer (4 sets of 4-min of alternated single leg work intervals) as aerobic exercise. The results revealed positive effects on ACD only after the combined exercise program, and it might indicate an important local exercise effect related to muscle mass quantity. In PAD patients, pulmonary oxygen kinetics were found to be slower in the lower extremities compared to upper extremities (Bauer et al., 2004) and circulatory adaptations were different when dynamic single-leg exercise is compared to two-leg exercise. This is related to the large redistribution of blood to the working muscles when a single-leg is used during exercise (Klausen et al., 1982). Another fact is that in Mosti et al. (2011) study, the authors combined aerobic exercise with 4 sets of 5 reps at 85–90% 1RM of dynamic leg press strength exercise, that represents a higher intensity and dose when compared to the other studies. Also, a meta-analysis found that higher intensity resistance exercise improves both flat ground and graded treadmill-walking performance in PAD patients (Parmenter et al., 2019).

The combined aerobic and resistance exercise compared to usual care promoted significant improvements in walking performance, but to date, when compared to treadmill walking, it seems to add no extra benefit to these variables. To the best of our knowledge, this is the first systematic review to compare the effects of combined aerobic and resistance exercise with isolated aerobic exercise or with usual care approach, providing important data regarding its impact on walking performance in PAD patients with IC.

### Limitations

The key limitations of this systematic review were the reduced number of Randomized Controlled Trials (the trials included in this systematic review had a moderate-to-high risk of bias), one of them was not a RCT, and there are some missing details in the protocol description.

## Conclusion

There is insufficient evidence to compare the effects on walking performance of combined aerobic and resistance exercise compared to isolated treadmill-walking or usual care approaches. However, despite the low quality of evidence, the combined aerobic and resistance exercise and isolated aerobic exercise (treadmill-walking) may lead to improve walking performance outcomes, such as COD, ACD, and MWD. Both reveal significant and positive results on walking performance; however, the combined exercise program may induce improvements in other important variables that were not the study target of this systematic review. The usual care approach has a trend to deteriorate walking performance. Thus, given the scarcity of data, high-quality RCTs that include an assessment of cardiovascular risk factors are urgently required to determine the effect of this exercise dose combination.

### Recommendations for Future Research

This systematic review combined the results of 337 participants with seven studies, indicating a small sample size and a small number of articles included. Therefore, more randomized controlled trials are needed to make a meaningful comparison between combined aerobic and resistance exercise programs and, a new review should be conducted, including a meta-analysis, where possible. Additional outcomes to quantity functional lower limb strength, cardiovascular risk factors, and qualitative data should be implemented. The exercise programs applied should carefully describe the key components of training protocols, specifying the mode of exercise, intensity, week frequency, exercise duration, and the exercise load progression, since this was not always clear.

### Practice Recommendations

Compared to usual care approach, short-term combined aerobic and resistance exercise programs and isolated aerobic exercise are essential for better improvements in walking performance (e.g., claudication onset distance and absolute claudication distance) in patients with intermittent claudication and peripheral arterial disease.

## Data Availability Statement

All datasets generated for this study are included in the article/Supplementary Material.

## Author Contributions

The literature search and selection of studies was performed by authors IM and CA. Following an initial screen of titles and abstracts (IM), full scrutiny of potentially eligible studies was independently screened by IM and CA using the specific inclusion criteria. NS arbitrated any disagreements in the study inclusion. Study quality assessment was performed by IM. CA, NS, HP, and JF revised the manuscript. All authors contributed to the development of the final manuscript, reviewed, and approved the submitted version.

### Conflict of Interest

The authors declare that the research was conducted in the absence of any commercial or financial relationships that could be construed as a potential conflict of interest.

## References

[B1] Aboyans V. Ricco J. B. Bartelink M. E. L. Bjorck M. Brodmann M. Cohnert T. (2018). 2017 ESC guidelines on the diagnosis and treatment of peripheral arterial diseases, in collaboration with the European society for vascular surgery (ESVS). Eur. J. Vasc. Endovasc. Surg. 55, 305–368. 10.1016/j.ejvs.2017.07.018 28851596

[B2] Baker W. B. Li Z. Schenkel S. S. Chandra M. Busch D. R. Englund E. K.. (2017). Effects of exercise training on calf muscle oxygen extraction and blood flow in patients with peripheral artery disease. J. Appl. Physiol. 123, 1599–1609. 10.1152/japplphysiol.00585.2017 28982943 PMC5814687

[B3] Bauer T. A. Brass E. P. Nehler M. Barstow T. J. Hiatt W. R. (2004). Pulmonary VO2 dynamics during treadmill and arm exercise in peripheral arterial disease. J. Appl. Physiol. 97, 627–634. 10.1152/japplphysiol.00612.2003 15090483

[B4] Currie K. D. Bailey K. J. Jung M. E. McKelvie R. S. MacDonald M. J. (2015). Effects of resistance training combined with moderate-intensity endurance or low-volume high-intensity interval exercise on cardiovascular risk factors in patients with coronary artery disease. J. Sci. Med. Sport 18, 637–642. 10.1016/j.jsams.2014.09.013 25308628

[B5] Delaney C. L. Miller M. D. Chataway T. K. Spark J. I. (2014). A randomised controlled trial of supervised exercise regimens and their impact on walking performance, skeletal muscle mass and calpain activity in patients with intermittent claudication. Eur. J. Vasc. Endovasc. Surg. 47, 304–310. 10.1016/j.ejvs.2013.12.021 24445084

[B6] Gardner A. W. Parker D. E. Montgomery P. S. Blevins S. M. (2014). Step-monitored home exercise improves ambulation, vascular function, and inflammation in symptomatic patients with peripheral artery disease: a randomized controlled trial. J. Am. Heart Assoc. 3:e001107. 10.1161/JAHA.114.001107 25237048 PMC4323792

[B7] Gerhard-Herman M. D. Gornik H. L. Barrett C. Barshes N. R. Corriere M. A. Drachman D. E. (2017). 2016 AHA/ACC guideline on the management of patients with lower extremity peripheral artery disease: a report of the American College of Cardiology/American Heart Association Task Force on clinical practice guidelines. Circulation 135, e726–e779. 10.1161/CIR.0000000000000470 27840333 PMC5477786

[B8] Golledge J. Singh T. P. Alahakoon C. Pinchbeck J. Yip L. Moxon J. V.. (2019). Meta-analysis of clinical trials examining the benefit of structured home exercise in patients with peripheral artery disease. Br. J. Surg. 106, 319–331. 10.1002/bjs.11101 30791089

[B9] Gonzalez A. M. Mangine G. T. Fragala M. S. Stout J. R. Beyer K. S. Bohner J. D.. (2014). Resistance training improves single leg stance performance in older adults. Aging Clin. Exp. Res. 26, 89–92. 10.1007/s40520-013-0126-6 23959961

[B10] Hamburg N. M. Balady G. J. (2011). Exercise rehabilitation in peripheral artery disease: functional impact and mechanisms of benefits. Circulation 123, 87–97. 10.1161/CIRCULATIONAHA.109.881888 21200015 PMC3061490

[B11] Hiatt W. R. Wolfel E. E. Meier R. H. Regensteiner J. G. (1994). Superiority of treadmill walking exercise versus strength training for patients with peripheral arterial disease. Implications for the mechanism of the training response. Circulation 90, 1866–1874. 10.1161/01.cir.90.4.1866 7923674

[B12] Jakubseviciene E. Vasiliauskas D. Velicka L. Kubilius R. Milinaviciene E. Vencloviene J. (2014). Effectiveness of a new exercise program after lower limb arterial blood flow surgery in patients with peripheral arterial disease: a randomized clinical trial. Int. J. Environ. Res. Public Health 11, 7961–7976. 10.3390/ijerph110807961 25105547 PMC4143843

[B13] Klausen K. Secher N. H. Clausen J. P. Hartling O. Trap-Jensen J. (1982). Central and regional circulatory adaptations to one-leg training. J. Appl. Physiol. Respir. Environ. Exerc. Physiol. 52, 976–983. 10.1152/jappl.1982.52.4.976 7085432

[B14] Kraus W. E. Powell K. E. Haskell W. L. Janz K. F. Campbell W. W. Jakicic J. M.. (2019). Physical activity, all-cause and cardiovascular mortality, and cardiovascular disease. Med. Sci. Sports Exerc. 51, 1270–1281. 10.1249/MSS.0000000000001939 31095084 PMC6527136

[B15] Kropielnicka K. Dziubek W. Bulinska K. Stefanska M. Wojcieszczyk-Latos J. Jasinski R.. (2018). Influence of the physical training on muscle function and walking distance in symptomatic peripheral arterial disease in elderly. Biomed. Res. Int. 2018:1937527. 10.1155/2018/1937527 30345295 PMC6174806

[B16] Lane R. Harwood A. Watson L. Leng G. C. (2017). Exercise for intermittent claudication. Cochrane Database Syst. Rev. 12:CD000990. 10.1002/14651858.CD000990.pub4 29278423 PMC6486315

[B17] Lauret G. J. Fakhry F. Fokkenrood H. J. Hunink M. G. Teijink J. A. Spronk S. (2014). Modes of exercise training for intermittent claudication. Cochrane Database Syst. Rev 7:CD009638 10.1002/14651858.CD009638.pub2 24993079

[B18] Leeper N. J. Myers J. Zhou M. Nead K. T. Syed A. Kojima Y.. (2013). Exercise capacity is the strongest predictor of mortality in patients with peripheral arterial disease. J. Vasc. Surg. 57, 728–733. 10.1016/j.jvs.2012.07.051 23044259 PMC3543469

[B19] Liberati A. Altman D. G. Tetzlaff J. Mulrow C. Gøtzsche P. C. Ioannidis J. P.. (2009). The PRISMA statement for reporting systematic reviews and meta-analyses of studies that evaluate health care interventions: explanation and elaboration. PLoS Med. 6:e1000100. 10.1371/journal.pmed.1000100 19621070 PMC2707010

[B20] Lima A. Correia M. A. Soares A. H. G. Farah B. Q. Forjaz C. L. M. Silva A. S.. (2018). Acute effects of walking and combined exercise on oxidative stress and vascular function in peripheral artery disease. Clin. Physiol. Funct. Imaging 38, 69–75. 10.1111/cpf.12384 27491344

[B21] Mays R. J. Regensteiner J. G. (2013). Exercise therapy for claudication: latest advances. Curr. Treat. Options Cardiovasc. Med. 15, 188–199. 10.1007/s11936-013-0231-z 23436041 PMC3627476

[B22] McDermott M. M. (2013). Functional impairment in peripheral artery disease and how to improve it in 2013. Curr. Cardiol. Rep. 15:347. 10.1007/s11886-013-0347-5 23420443 PMC3683561

[B23] McDermott M. M. Ades P. Guralnik J. M. Dyer A. Ferrucci L. Liu K.. (2009). Treadmill exercise and resistance training in patients with peripheral arterial disease with and without intermittent claudication: a randomized controlled trial. JAMA 301, 165–174. 10.1001/jama.2008.962 19141764 PMC3268032

[B24] McDermott M. M. Guralnik J. M. Criqui M. H. Liu K. Kibbe M. R. Ferrucci L. (2014). Six-minute walk is a better outcome measure than treadmill walking tests in therapeutic trials of patients with peripheral artery disease. Circulation 130, 61–68. 10.1161/CIRCULATIONAHA.114.007002 24982117 PMC4154227

[B25] McDermott M. M. Guralnik J. M. Ferrucci L. Tian L. Kibbe M. R. Greenland P.. (2016). Community walking speed, sedentary or lying down time, and mortality in peripheral artery disease. Vasc. Med. 21, 120–129. 10.1177/1358863X15626521 26873873 PMC5656391

[B26] Moher D. Liberati A. Tetzlaff J. Altman D. G. (2009). Preferred reporting items for systematic reviews and meta-analyses: the PRISMA statement. PLoS Med. 6:e1000097 10.1371/journal.pmed.1000097 19621072 PMC2707599

[B27] Morris D. R. Rodriguez A. J. Moxon J. V. Cunningham M. A. McDermott M. M. Myers J.. (2014). Association of lower extremity performance with cardiovascular and all-cause mortality in patients with peripheral artery disease: a systematic review and meta-analysis. J. Am. Heart Assoc. 3:e001105. 10.1161/JAHA.114.001105 25122666 PMC4310407

[B28] Mosti M. P. Wang E. Wiggen O. N. Helgerud J. Hoff J. (2011). Concurrent strength and endurance training improves physical capacity in patients with peripheral arterial disease. Scand. J. Med. Sci. Sports 21, e308–e314. 10.1111/j.1600-0838.2011.01294.x 21410546

[B29] Nicolai S. P. Viechtbauer W. Kruidenier L. M. Candel M. J. Prins M. H. Teijink J. A. (2009). Reliability of treadmill testing in peripheral arterial disease: a meta-regression analysis. J. Vasc. Surg. 50, 322–329. 10.1016/j.jvs.2009.01.042 19631868

[B30] Nordanstig J. Broeren M. Hensater M. Perlander A. Osterberg K. Jivegard L. (2014). Six-minute walk test closely correlates to “real-life” outdoor walking capacity and quality of life in patients with intermittent claudication. J. Vasc. Surg. 60, 404–409. 10.1016/j.jvs.2014.03.003 24690492

[B31] Novakovic M. Jug B. Lenasi H. (2017). Clinical impact of exercise in patients with peripheral arterial disease. Vascular 25, 412–422. 10.1177/1708538116678752 28256934

[B32] Papa E. V. Dong X. Hassan M. (2017). Resistance training for activity limitations in older adults with skeletal muscle function deficits: a systematic review. Clin. Interv. Aging 12, 955–961. 10.2147/CIA.S104674 28670114 PMC5479297

[B33] Parmenter B. J. Mavros Y. Ritti Dias R. King S. Fiatarone Singh M. (2019). Resistance training as a treatment for older persons with peripheral artery disease: a systematic review and meta-analysis. Br. J. Sports Med. 10.1136/bjsports-2018-100205. [Epub ahead of print]. 30979698

[B34] Parr B. M. Noakes T. D. Derman E. W. (2009). Peripheral arterial disease and intermittent claudication: efficacy of short-term upper body strength training, dynamic exercise training, and advice to exercise at home. S. Afr. Med. J. 99, 800–804. 20218480

[B35] Pedersen M. T. Vorup J. Nistrup A. Wikman J. M. Alstrom J. M. Melcher P. S.. (2017). Effect of team sports and resistance training on physical function, quality of life, and motivation in older adults. Scand. J. Med. Sci. Sports 27, 852–864. 10.1111/sms.12823 28144978

[B36] Piercy K. L. Troiano R. P. Ballard R. M. Carlson S. A. Fulton J. E. Galuska D. A.. (2018). The physical activity guidelines for Americans. JAMA 320, 2020–2028. 10.1001/jama.2018.14854 30418471 PMC9582631

[B37] Ritti-Dias R. M. Wolosker N. de Moraes Forjaz C. L. Carvalho C. R. Cucato G. G. Leao P. P.. (2010). Strength training increases walking tolerance in intermittent claudication patients: randomized trial. J. Vasc. Surg. 51, 89–95. 10.1016/j.jvs.2009.07.118 19837534

[B38] Schroeder E. C. Franke W. D. Sharp R. L. Lee D. C. (2019). Comparative effectiveness of aerobic, resistance, and combined training on cardiovascular disease risk factors: a randomized controlled trial. PLoS ONE 14:e0210292. 10.1371/journal.pone.0210292 30615666 PMC6322789

[B39] Silva Rde C. Wolosker N. Yugar-Toledo J. C. Consolim-Colombo F. M. (2015). Vascular Reactivity is Impaired and Associated With Walking Ability in Patients With Intermittent Claudication. Angiology 66, 680–686. 10.1177/0003319714545486 25100750

[B40] Sousa N. Mendes R. Abrantes C. Sampaio J. Oliveira J. (2013). Long-term effects of aerobic training versus combined aerobic and resistance training in modifying cardiovascular disease risk factors in healthy elderly men. Geriatr. Gerontol. Int. 13, 928–935. 10.1111/ggi.12033 23441809

[B41] Stewart K. J. Hiatt W. R. Regensteiner J. G. Hirsch A. T. (2002). Exercise training for claudication. N. Engl. J. Med. 347, 1941–1951. 10.1056/NEJMra021135 12477945

[B42] Tebbutt N. Robinson L. Todhunter J. Jonker L. (2011). A plantar flexion device exercise programme for patients with peripheral arterial disease: a randomised prospective feasibility study. Physiotherapy 97, 244–249. 10.1016/j.physio.2010.08.009 21820543

[B43] Vascular Disease Foundation (2012). “PAD exercise training toolkit: a guide for health care professionals. Healthy steps for peripheral artery disease,” in Helthy Steps for Peripheral Artery Disease (Vienna, VA: American Association of Cardiovascular and Pulmonary Rehabilitation), 37.

[B44] Wang E. Helgerud J. Loe H. Indseth K. Kaehler N. Hoff J. (2010). Maximal strength training improves walking performance in peripheral arterial disease patients. Scand. J. Med. Sci. Sports 20, 764–770. 10.1111/j.1600-0838.2009.01014.x 19804581

[B45] Winzer E. B. Woitek F. Linke A. (2018). Physical activity in the prevention and treatment of coronary artery disease. J. Am. Heart Assoc. 7:e007725. 10.1161/JAHA.117.007725 29437600 PMC5850195

[B46] Yucel H. Turkdogan K. A. Zorlu A. Aydin H. Kurt R. Yilmaz M. B. (2015). Association between oxidative stress index and post-CPR early mortality in cardiac arrest patients: a prospective observational study. Anatol. J. Cardiol. 15, 737–743. 10.5152/akd.2014.5719 25592095 PMC5368483

[B47] Zhang Y. Fischer K. E. Soto V. Liu Y. Sosnowska D. Richardson A.. (2015). Obesity-induced oxidative stress, accelerated functional decline with age and increased mortality in mice. Arch. Biochem. Biophys. 576, 39–48. 10.1016/j.abb.2014.12.018 25558793 PMC4456198

[B48] Zhang Y. Qi L. Xu L. Sun X. Liu W. Zhou S.. (2018). Effects of exercise modalities on central hemodynamics, arterial stiffness and cardiac function in cardiovascular disease: Systematic review and meta-analysis of randomized controlled trials. PLoS ONE 13:e0200829. 10.1371/journal.pone.0200829 30036390 PMC6056055

